# The second known stygomorphic freshwater crab from China, *Phasmon typhlops* gen. nov. et sp. nov. (Crustacea, Decapoda, Potamidae), diverged at the beginning of the Late Miocene

**DOI:** 10.3897/zookeys.1008.58854

**Published:** 2020-12-31

**Authors:** Chao Huang, Shane T. Ahyong, Hsi-Te Shih

**Affiliations:** 1 Unaffiliated, Guangdong, China Unaffiliated Guangdong China; 2 Australian Museum, 1 William St, Sydney NSW 2010, Australia Australian Museum Sydney Australia; 3 Department of Life Science and Research Center for Global Change Biology, National Chung Hsing University, Taichung 402, Taiwan University of New South Wales Kensington Australia; 4 School of Biological, Earth and Environmental Sciences, University of New South Wales, Kensington, NSW 2052, Australia National Chung Hsing University Taichung Taiwan

**Keywords:** 16S rDNA, blind crab, cave crab, new genus, new species, Guangxi, subterranean

## Abstract

A new genus and new species of blind freshwater cave crab are described from Chongzuo City, Guangxi Zhuang Autonomous Region, China based on morphology and mitochondrial 16S rDNA sequences. The new genus, *Phasmon***gen. nov.**, is established for *P. typhlops***sp. nov.**, which is only the second blind cave crab known from China and East Asia. The combination of a very wide carapace, overall depigmentation, reduced orbits and vestigial unpigmented eyes of *Phasmon* immediately separates it from all known potamid genera. Molecular divergence estimates based on 16S rDNA suggest that the lineage to which the new genus belongs diverged from other potamids at the beginning of the Late Miocene (10.8 million years ago), much earlier than other Chinese cave crabs.

## Introduction

The Guangxi Zhuang Autonomous Region is located in southern China within the Huanan freshwater zoogeographic province (Huang et al. 2020a) and borders Vietnam. Its complex physical geography and subtropical climate make it one of the richest regions for freshwater crabs in China (Shih and Ng 2011; Chu et al. 2018). With 14 genera recorded to date (*Bottapotamon* Türkay & Dai, 1997, *Chinapotamon* Dai & Naiyanetr, 1994, *Heterochelamon* Türkay & Dai, 1997, *Indochinamon* Yeo & Ng, 2007, *Lacunipotamon* Dai, Song, He, Cao, Xu & Zhong, 1975, *Longpotamon* Shih, Huang & Ng, 2016, *Mediapotamon* Türkay & Dai, 1997, *Neilupotamon* Dai & Türkay, 1997, *Potamiscus* Alcock, 1909, *Qianguimon* Huang, 2018, *Sinolapotamon* Tai & Sung, 1975, *Somanniathelphusa* Bott, 1968, *Tiwaripotamon* Bott, 1970, and *Yarepotamon* Dai & Türkay, 1997), Guangxi Zhuang Autonomous Region is second, in China, only to Yunnan Province in the number of freshwater crab genera (Shih and Ng 2011; Huang 2018; Huang et al. 2020d). From the number of new species described from this region in recent years (Zhu et al. 2010; Naruse et al. 2013; Do et al. 2016; Ng 2017; Huang 2018; Zou et al. 2018; Wang et al. 2019, 2020a, b), there is little doubt that many more remain to be discovered.

Stygomorphic potamid crabs are rare, and only a few species were previously known. *Chinapotamon clarkei* Ng, 2017 and *Diyutamon cereum* Huang, Shih & Ng, 2017b, from China; *Cerberusa tipula* Holthuis, 1979 and *Cerberusa caeca* Holthuis, 1979, from Borneo; *Erebusa calobates* Yeo & Ng, 1999, from Laos; and *Teretamon spelaeum* Absar, Mitra & Kharkongor, 2017, from India, all – exhibit varying degrees of stygomorphism. Of these, only *D. cereum* and *C. caeca* exhibit strong eye reduction and depigmentation–both appear to be blind and can be considered the most highly stygomorphic of known subterranean potamids.

In 2018, a local collector from Chongzuo City, Guangxi, China, alerted us to an unusual crab trapped from a karst spring. This crab, a female, was apparently a stygobite, lacking body pigmentation and having vestigial, unpigmented eyes. Many further attempts were made to collect more specimens of this unusual species, but they remained elusive. The collector only succeeded once in trapping a second specimen from the karst spring, but only half of the carcass remained when he checked the trap; the other half was apparently consumed by a specimen of the epigean crab *Lacunipotamon cymatile* Huang, Shih & Ahyong, 2020 (Huang et al. 2020c), which was also lured into the trap. It was not until over a year later that the collector discovered the primary habitat of the crab in a nearby cave system in which he successfully trapped more specimens. After we acquired and examined the specimens, it was obvious that these cave crabs were new to science: the combination of highly reduced and unpigmented eyes, long antennules, lack of body pigmentation, unique carapace features, and very wide male anterior thoracic sternum, immediately separate it from all other potamid genera. Molecular data derived from the mitochondrial 16S rDNA gene further support the taxon as new, and we herein describe it as a new genus and new species.

## Materials and methods

Specimens were collected by hand, preserved in 75% ethanol, and deposited in the collections of the Sun Yat-sen Museum of Biology, Sun Yat-sen University, Guangzhou, China (SYSBM) and the Australian Museum, Sydney, Australia (AM). The terminology used primarily follows that of Dai (1999) and Davie et al. (2015). Carapace length (CL) was measured along the dorsal midline and carapace width (CW) was the greatest width measured across the branchial margins. The male gonopods 1 and 2 are abbreviated as G1 and G2, respectively. Measurements (mm) are of the carapace width and length, respectively.

A 16S sequence was obtained from the paratype (AM P.105524) following Shih et al. (2009), using the primers 16H10 and 16L29 (Schubart 2009), and aligned with the MUSCLE function of MEGA (vers. 10.0.5; Kumar et al. 2018) after verification with the complimentary strand. The sequence was deposited in NCBI GenBank under the accession number MW289910. A preliminary analysis showed that this genus belongs to the “China-East Asia Islands” Group within the “Eastern-Asia Subclade” of the subfamily Potamiscinae (Shih et al. 2009). Therefore, to confirm the phylogenetic position of the new genus and species, 24 additional 16S sequences from related genera from East Asia, Indochina and Southeast Asia in Shih et al. (2009), Huang et al. (2014, 2017a, b, 2018, 2020d), and Wang et al. (2019) were included for comparison. The variable regions in the loop regions of 16S that could not be aligned adequately for phylogenetic analysis were excluded (Shih et al. 2009).

The best-fitting model for sequence evolution of 16S was determined by PartitionFinder (vers. 2.1.1; Lanfear et al. 2017), selected by the Bayesian information criterion (BIC). The best model obtained, GTR+I+G, was subsequently applied for Bayesian inference (BI) and maximum likelihood (ML) analyses. The BI analysis was performed with MrBayes (vers. 3.2.2; Ronquist et al. 2012) using four chains run for 10 million generations, with trees sampled every 1000 generations. The convergence of chains was determined by the average standard deviation of split frequency values below the recommended 0.01 (Ronquist et al. 2005) and the first 1050 trees were discarded as burn-in accordingly. The ML analysis was conducted in RAxML (vers. 7.2.6; Stamatakis 2006). The GTR + G (i.e., GTRGAMMA) model was used for all subsets with 100 runs and found the best ML tree by comparing likelihood scores. The robustness of the ML tree was evaluated by 1000 bootstrap pseudoreplicates under the model GTRGAMMA. The uncorrected p-distances for genetic divergence between haplotypes were calculated by MEGA.

## Taxonomy

### Family Potamidae Ortmann, 1896

**Subfamily Potamiscinae Bott, 1970**

#### Phasmon gen. nov.

Taxon classificationAnimaliaDecapodaPotamidae

DA3B111E-120A-5378-9C24-3B2930BD38BD

http://zoobank.org/0BD28B59-9BE1-4679-AD9C-268006C41E77

Figs 1 , 2 , 3 , 4

##### Type species.

*Phasmon typhlops*, by present designation.

##### Diagnosis.

**Figure 1.**
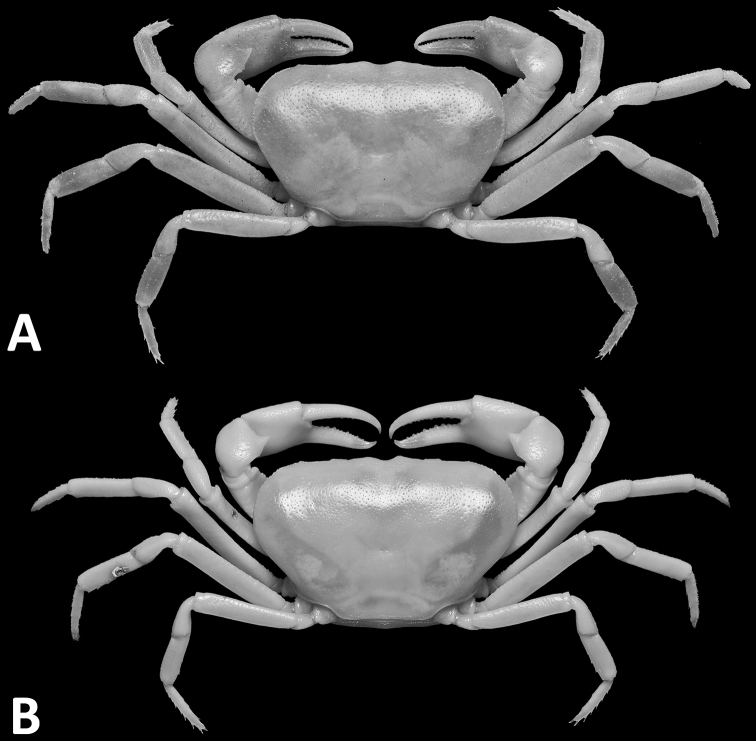
*Phasmon typhlops* gen. nov. et sp. nov., male holotype (14.4 × 9.0 mm), SYSBM 001982 (**A**) female paratype (22.1 × 13.7 mm), AM P.105524 (**B**). Dorsal habitus.

**Figure 2.**
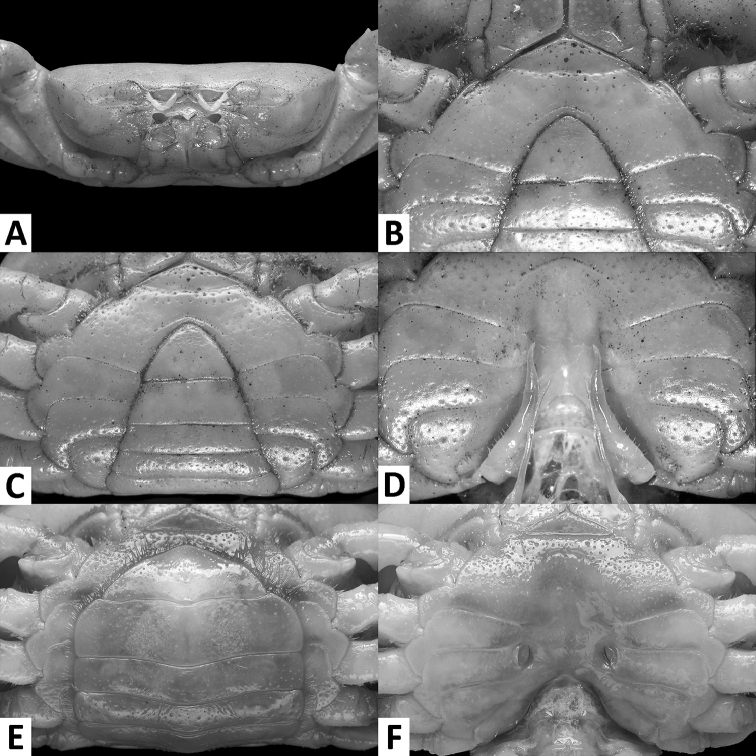
*Phasmon typhlops* gen. nov. et sp. nov., male holotype (14.4 × 9.0 mm), SYSBM 001982 (**A–D**) female paratype (22.1 × 13.7 mm), AM P.105524 (**E, F**). Cephalothorax, anterior view (**A**) anterior thoracic sternum (**B**) anterior thoracic sternum and pleon, ventral view (**C**) sterno-pleonal cavity with G1 *in situ*, ventral view (**D**) pleon, ventral view (**E**) vulvae, ventral view (**F**).

**Figure 3.**
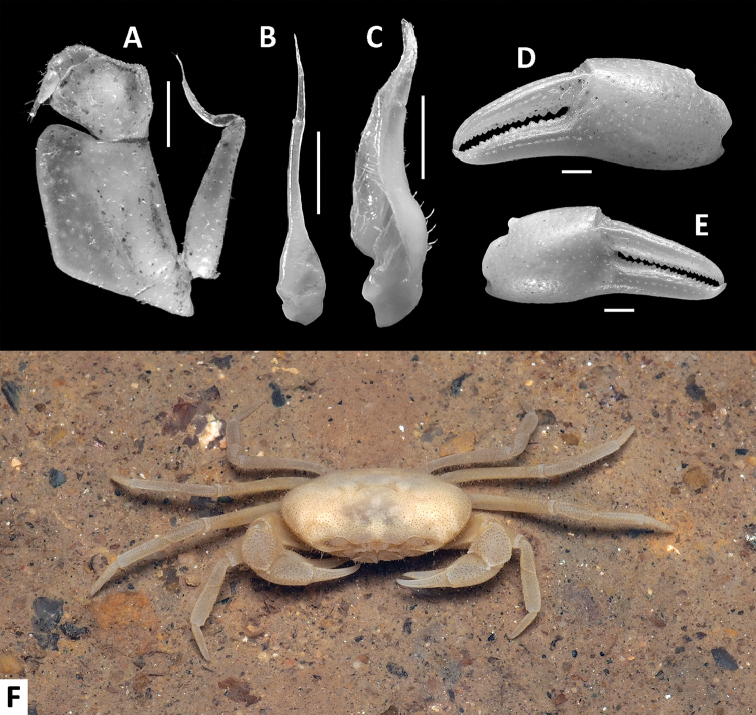
*Phasmon typhlops* gen. nov. et sp. nov., male holotype (14.4 × 9.0 mm), SYSBM 001982. Left maxilliped 3 (**A**) left G2, pleonal view (**B**) left G1, pleonal view (**C**) major cheliped (**D**) minor cheliped (**E**) colour in life (**F**). Scale bars: 1.0 mm.

**Figure 4.**
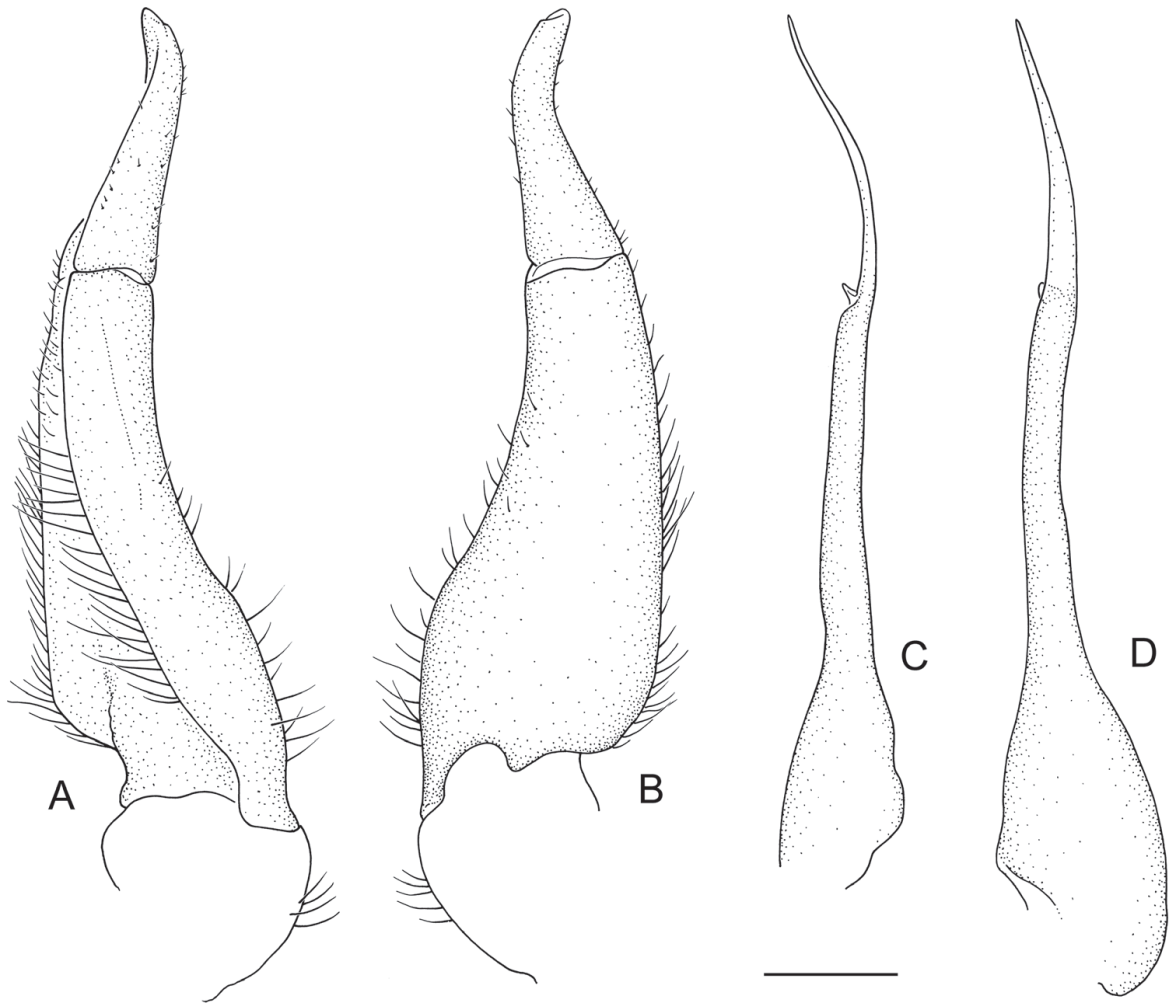
*Phasmon typhlops* gen. nov. et sp. nov., gonopods: left G1, abdominal view (**A**) left G1, sternal view (**B**) left G2, mesial view (**C**) left G2, pleonal view (**D**). Scale bar: 0.5 mm.

Small sized (carapace width less than 30 mm). Carapace 1.6× wider than long; fronto-orbital width about twice width of posterior margin; dorsal surface weakly convex (Figs 1, 2A); frontal margin weakly sinuous, continuous with supraorbital margin, forming almost straight anterior margin of carapace in dorsal view (Fig. 1); postorbital and epigastric cristae almost indiscernible (Figs 1, 2A); orbit shallow, eyes vestigial, almost immovable, length about half orbital width; cornea reduced, unpigmented, facets absent; external orbital angle very wide, confluent with anterolateral margin (Figs 1, 2A). Epibranchial tooth inconspicuous. Median lobe of epistome broadly triangular (Fig. 2A). Maxilliped 3 ischium length less than twice width; exopod reaching beyond distal edge of ischium, flagellum well-developed (Fig. 3A). Cheliped fingers without gape when closed (Fig. 3D, E). Male anterior thoracic sternum very wide, around 2.3 times as wide as long (Fig. 2B). Male pleon triangular (Fig. 2C). G1 tapering anteriorly, tip narrow but blunt (Figs 3C, 4A, B). G2 distal segment tip pointed (Figs 3B, 4C, D). Female vulvae on sternite 6, reaching sutures of sternites 5/6 anteriorly, very widely spaced from one another (Fig. 2F).

##### Etymology.

The genus name is an arbitrary combination of the Latin word “phasma”, meaning ghost, which refers to the type species’ pale appearance and dark habitat, and the genus name *Potamon*, which is the type genus of the family. Gender neuter.

#### Phasmon typhlops sp. nov.

Taxon classificationAnimaliaDecapodaPotamidae

DB076607-29E0-5031-B657-44A6C14D4B3D

http://zoobank.org/FEE83A41-2669-4B3F-B3A0-5E1F15F1DEB1

Figs 1 , 2 , 3 , 4

##### Type material.

***Holotype***: SYSBM 001982, male (14.4 × 9.0 mm), Leiping Town, Daxin County, Chongzuo City, Guangxi Province, China, 22.65°N, 107.10°E, subterranean karst stream in cave, baited trap, coll. local collector, December 2019. ***Paratype***: AM P.105524, female (22.1×13.7 mm), Leiping Town, Daxin County, Chongzuo City, Guangxi Province, China, karst spring, baited trap, coll. local collector, September 2018.

##### Description.

Carapace broad, about 1.6 times as wide as long; fronto-orbital width about twice width of posterior margin; regions indistinct, dorsal surface slightly convex; surface finely pitted (Fig. 1A). Frontal margin weakly sinuous, continuous with supraorbital margin, forming almost straight transverse margin in dorsal view (Figs 1A, B). Epigastric cristae and postorbital cristae almost indiscernible (Figs 1A, 2A). Branchial regions slightly swollen (Figs 1A, 2A). Cervical groove shallow (Fig. 1A). Mesogastric region slightly convex (Fig. 1A). External orbital angle obsolete, outer margin convex, almost indistinguishable from anterolateral margin (Figs 1A, 2A). Epibranchial tooth granular, inconspicuous (Fig. 1A). Anterolateral margin lined with 15–20 small, single or partially fused granules. Posterolateral margin posteriorly convergent (Fig. 1A); posterolateral surface generally smooth (Fig. 1A). Orbits shallow; supraorbital margins weakly cristate, infraorbital margins lined with granules (Fig. 2A). Eyes almost immobile, greatly reduced, tapering, length about half orbital width; peduncle short, stout; cornea vestigial, surface without facets, unpigmented (Figs 2A, 3F). Sub-orbital, pterygostomial and sub-hepatic regions generally smooth, pitted (Fig. 2A). Antennules large, folded within broad fossae; antennae very short (Fig. 2A). Median lobe of epistome posterior margin broadly triangular, lateral margins sinuous (Fig. 2A).

Maxilliped 3 merus subtrapezoidal, with median depression, width about 1.2× length; ischium subtrapezoidal with shallow median sulcus, distomesial margin rounded, width about 0.6× length. Exopod reaching proximal one-third of merus; flagellum longer than half ischium length (Fig. 3A).

Chelipeds (pereiopod 1) subequal (Figs 1, 3D, E). Merus trigonal in cross section; margins slightly crenulated, surface generally smooth (Figs 1A, 2A). Carpus with sharp spine at inner-distal angle (Fig. 1 A). Major cheliped palm length about 1.5× height; dactylus 0.9× palm length (male) (Fig. 3D, E), as long as palm (female). Palm surface pitted (Fig. 3D, E). Dactylus as long as pollex (Fig. 3D, E). Occlusal margin of fingers with 18–20 irregular blunt teeth, without gape when closed (Fig. 3D, E).

Ambulatory legs (pereiopods 2–5) slender with very sparse short setae (Fig. 1). Pereiopod 3 merus 0.9× CL (male) (Fig. 1A), 0.8× CL (female) (Fig. 1B). Pereiopod 5 propodus length 2.8× height (male) (Fig. 1A), 3.4 height (female) (Fig. 1B), shorter than dactylus; dactylus length 6.1× height (male) (Fig. 1A), 6.2× height (female) (Fig. 1B).

Male thoracic sternum generally smooth, pitted; sternites 1–4 width about 2.3× length; sternites 1, 2 forming indistinguishably fused, broad triangle; fused sternites 1, 2 demarcated from sternite 3 by shallow transverse sulcus; sternites 3, 4 fused without indication of demarcation except for shallow lateral notch (Fig. 2B). Male sterno-pleonal cavity reaching anteriorly slightly beyond level of cheliped coxa articular condyle (Fig. 2B); deep median longitudinal groove between sternites 7, 8 (Fig. 2D). Male pleonal locking tubercle positioned at mid-length of sternite 5 (Fig. 2D). Female vulvae reaching sutures of sternites 5/6 anteriorly but not posteriorly to sutures of sternites 6/7, positioned widely apart from each other (Fig. 2F).

Male pleon broadly triangular; somites 3–6 progressively narrower; somite 6 width approximately 2.7× length; telson width 1.6× length; lateral margins slightly convex, apex rounded (Fig. 2C). Female pleon subovate (Fig. 2E).

G1 tapering, slightly sinuous, tip exceeding pleonal locking tubercle but not reaching suture between thoracic sternites 4/5 *in situ* (Fig. 2D); proximal segment length about 2.3× length of distal segment (Figs 3C, 4A, B). Distal segment slender, tapering anteriorly, slightly inclined towards midline; tip pointed upwards in dissected view (Figs 3C, 4A, B). G2 slender, almost straight, proximal portion with distal two-thirds subcylindrical, length about 2.4× length of distal portion (Figs 3B, 4C, D); distal portion flattened, apex acute, proximally with small triangular lobe.

##### Etymology.

The species name is derived from the Greek words “typhlos” and “ops”, meaning “blind” and “eyes”, respectively. It refers to the greatly reduced and non-functional eyes of this species.

##### Colour in life.

Pale yellowish-white all over (Fig. 3F).

##### Habitat.

*Phasmon typhlops* gen. nov. et sp. nov. occurs in subterranean karst streams, but little is currently known about its precise habitat. According to the collector, subterranean streams in the dark zone of caves appear to be the primary habitat of *P. typhlops* sp. nov., where it has been found in shallow and still water as well as flowing streamways. However, some specimens have also been captured at night from a karstic spring that is immediately connected to the more extensive subterranean karst system. We only examined the two type specimens, of which the holotype was collected from the former habitat and the paratype from the latter. An epigean species, *Lacunipotamon cymatile*, inhabits the areas immediately adjacent to the spring and has been observed to prey on *Phasmon typhlops* gen. nov. et sp. nov. (Huang et al. 2020c).

##### Distribution.

Chongzuo City, Guangxi Province, China.

##### Remarks.

*Phasmon typhlops* gen. nov. et sp. nov. can be considered a true stygobite owing to its stygomorphic features, in particular the strong reduction of the eyes, body depigmentation and slightly elongated appendages, which are consistent with its subterranean lifestyle (Holthuis 1986; Ng and Goh 1987). Apart from *P. typhlops* gen. nov. et sp. nov., *Diyutamon cereum* and *Cerberusa caeca* are the only other apparently blind stygomorphic potamid crabs known. We have not directly examined the eyes of *C. caeca*, but those of *D. cereum* and *P. typhlops* are unpigmented and the cornea is vestigial and without facets. Although we cannot exclude the possibility that the eyes of *D. cereum* and *P. typhlops* are capable of light detection, the absence of pigmentation or ommatidial facets indicates that the eyes are incapable of image formation. The enlarged antennules as present in *Phasmon* gen. nov. are otherwise seen in only a few cavernicolous freshwater crabs such as the gecarcinucids *Sundathelphusa waray* Husana, Naruse & Kase, 2009, and *S. lobo* Husana, Naruse & Kase, 2009 (Husana et al. 2009: figs 2B, 5B), and are likely a sensory compensation for the loss of vision (Culver et al. 1995). Other than these two species, there are other stygomorphic gecarcinucids from Asia, but these can be separated from the new species by obvious family-level characters.

Sexual dimorphism is evident in our two specimens of *P. typhlops*: the smaller male holotype has proportionally longer but stouter legs in comparison to the larger female. The anterior carapace of the larger female is also proportionately wider than the posterior than in the male. Although the differences in leg proportions follow the pattern of sexual dimorphism observed in other potamids (e.g., Huang et al. 2020b), whether this carapace difference is due to size, sex or general variation remains to be determined.

Taxonomically, the most striking features of *Phasmon* gen. nov. are its very wide carapace (CW/CL=1.6; Fig. 1) and wide male anterior thoracic sternum (width 2.3× length; Fig. 2B). These characters combined immediately separate *Phasmon* gen. nov. from all other potamid genera. *Diyutamon cereum* occurs in Guizhou, which is relatively close to the type locality of *P. typhlops* gen. nov. et sp. nov. *Phasmon typhlops* gen. n. et sp. n. can be separated from *D. cereum* by its proportionally wider carapace (CW/CL=1.6 vs. 1.3–1.4 in *D. cereum*; Huang et al. 2017b: fig. 2A); granulate anterolateral carapace margins (Fig. 1) (vs. spinose in *D. cereum*; Huang et al. 2017b: fig. 2A); proportionally wider male anterior thoracic sternum (width 2.3× length vs. width 1.7× length in *D. cereum*; Huang et al. 2017b: fig. 6C); proportionally wider male pleon (compare Fig. 2C with Huang et al. 2017b: fig. 2C); male thoracic sternite 8 being fully concealed when the pleon is closed (Fig. 2C) (vs. partially exposed in *D. cereum*; Huang et al. 2017b: fig. 3E, F); and its relatively shorter and stouter walking legs (Fig. 1) (see Huang et al. 2017b: fig. 2A).

*Phasmon typhlops* gen. nov. et sp. nov. is similar to *Cerberusa caeca* in general physiognomy and size. However, the new species can immediately be distinguished by its proportionally wider carapace (CW/CL=1.6 vs. 1.3–1.4 in *C. caeca*; Holthuis 1979: pl. 8); almost indiscernible postorbital cristae (Fig. 1) (vs. low, indicated by a transverse row of granules in *C. caeca*; Holthuis 1979: fig. 3A); proportionally wider male pleon (compare Fig. 2C with Holthuis 1979: fig. 3C); and its slightly sinuous G1 (Figs 3C, 4A, B) (vs. strongly bent outwards in *C. caeca*; Holthuis 1979: fig. 3D).

The G1 characteristics of *Phasmon* gen. nov. are rather unremarkable and particularly similar to those of *Chinapotamon* and *Diyutamon*. *Chinapotamon* is also found in Guangxi and includes two cavernicolous species, *C. dashiwei* Ng, 2017 and *C. clarkei* Ng, 2017, of which the latter displays evidence of stygomorphism in reduced body pigmentation and well-developed, albeit proportionally smaller eyes than epigean congeners (Ng 2017). *Phasmon* gen. nov. is readily distinguished from *Chinapotamon* in: the proportionally wider carapace (CW/CL=1.6 vs. 1.3–1.4 in *Chinapotamon*; Ng 2017: figs 2, 6; Zou et al. 2018: fig. 2); the frontal margin being continuous with the supraorbital margin, forming an almost straight transverse margin in dorsal view (Fig. 1) (vs. supraorbital margin distinctly concave in dorsal view in *Chinapotamon*; Ng 2017: figs 2, 6; Zou et al. 2018: fig. 2); the vestigial, unpigmented eyes (Fig. 2A) (vs. well-developed, pigmented eyes in *Chinapotamon*; Ng 2017: fig. 6); the almost indiscernible epigastric cristae and postorbital cristae (Fig. 1) (vs. clearly discernible in *Chinapotamon*; Ng 2017: figs 2, 6; Zou et al. 2018: fig. 2); the proportionally wider male anterior thoracic sternum (width/length 2.3 vs. 1.6–1.7 in *Chinapotamon*; Ng 2017: figs 3A, 7A; Zou et al. 2018: fig. 3A); and the proportionally wider male pleon (compare Fig. 2C with Ng 2017: figs 3B, 7B; Zou et al. 2018: fig. 3B).

## DNA analyses and discussion

**Figure 5.**
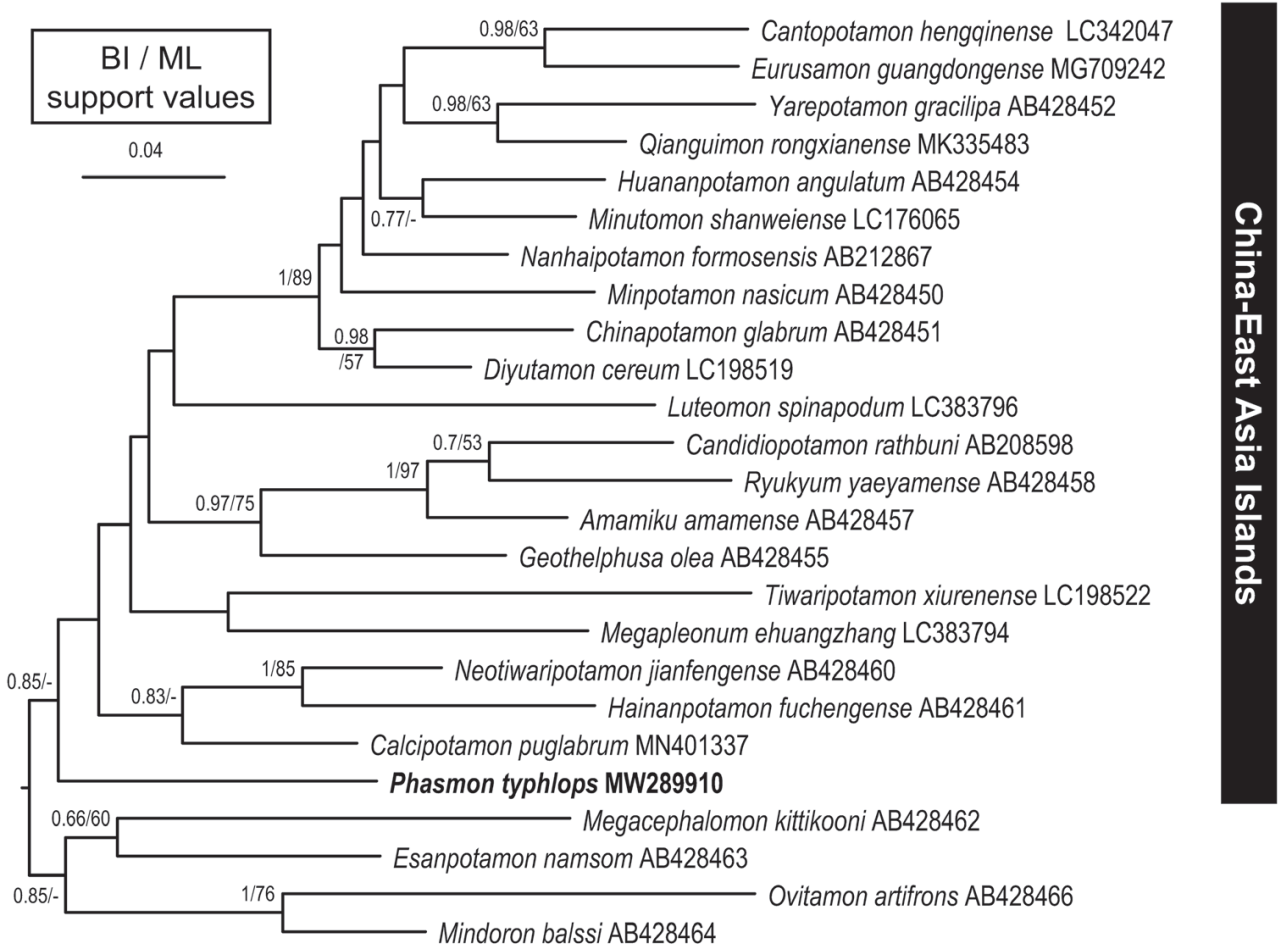
Bayesian inference (BI) tree of 16S rDNA for the “China-East Asian Islands” Group of the subfamily Potamiscinae. Support values at nodes represent posterior probabilities and bootstrap proportions > 50% for BI and maximum likelihood (ML), respectively.

A 512-basepair segment of the 16S rDNA gene, excluding the variable regions, was amplified and aligned from 25 potamid genera. The phylogenetic tree of the 16S sequences reconstructed using BI analysis is shown with support values from ML analysis (Fig. 5). The phylogenetic results place *Phasmon* gen. nov., albeit with weak support, in a basal position of the “China-East Asia Islands” Group. Although the sister group to *Phasmon* could not be robustly determined, *Phasmon* gen. nov. is clearly phylogenetically distant from the stygobitic *Diyutamon*, indicating that the two lineages independently colonized subterranean habitats. Treating *Phasmon* gen. nov. as a basal clade and applying the substitution rate of 0.88% for 16S rDNA for terrestrial *Sesarma* and other freshwater crabs (Schubart et al. 1998; Shih et al. 2006, 2009; Huang et al. 2017b), the divergence time between *Phasmon* gen. nov. and other genera in the “China-East Asia Islands” Group is estimated at 10.8 ± 1.0 mya (= million years ago) (with uncorrected p-distance of 9.49% ± 0.91%).

The divergence time of 10.8 mya estimated for *Phasmon* gen. nov. is much older than the 5.7 mya estimated for another Chinese cave crab genus, *Diyutamon* (Huang et al. 2017b). The karst caves of this region are estimated to have formed in the Pleistocene (2.58–0.0117 mya) based on animal and plant fossils (Mead et al. 2014; Yan et al. 2014; Li et al. 2020). If ancestral *P. typhlops* gen. nov. et sp. nov. entered subterranean karst caves at the beginning of the Late Miocene (11.63–5.333 mya), then previous age estimates of the regional karsts would be substantially too young. However, it seems also more likely that although the lineage to which *Phasmon* gen. nov. belongs diverged in the Late Miocene, the ancestors of *P. typhlops* gen. nov. et sp. nov. probably entered the subterranean environment after it was formed later in the Pleistocene. The present-day distribution of *Phasmon* gen. nov. is probably relictual, and given its isolated phylogenetic position in the “China-East Asia Islands” Group, *P. typhlops* possibly represents the last of an otherwise extinct lineage.

## Supplementary Material

XML Treatment for Phasmon

XML Treatment for Phasmon typhlops
